# Supplementary material for: Multireference Equation-of-Motion-Driven Similarity Renormalization Group for X‑ray Photoelectron Spectra
Source: J Chem Theory Comput. 2025 Nov 26;21(23):12094–109. doi: 10.1021/acs.jctc.5c01616 (PMC12874376; doi:10.1021/acs.jctc.5c01616)
Supplement: Supplementary file 1 [file ct5c01616_si_001.pdf]

Supporting Information: Multireference equation-of-motion driven similarity renormalization group for X-ray photoelectron spectra

Shuhang Li,[1] Zijun Zhao,[1] and Francesco A. Evangelista[1, a)]

Department of Chemistry and Cherry Emerson Center for Scientific Computation, Emory University, Atlanta, Georgia 30322, United States

[a)]Electronic mail: francesco.evangelista@emory.edu

I. CHOICE OF ACTIVE SPACE AND REFERENCE ENERGIES

TABLE S1. The active space and corresponding CASSCF energy (in E_h) used in the EOM-DSRG calculations for each molecule. The active space is specified by the number of core and active orbitals per irrep, listed in 'Cotton ordering'.

Molecule	Core	Active	Energy (E_h)
C_2H_4	[1,0,0,0,0,0,0,1]	[3,2,1,0,0,1,2,3]	-78.237527
CH_4	[1,0,0,0]	[4,0,2,2]	-40.310940
C_2H_2	[1,0,0,0,0,1,0,0]	[3,0,1,1,0,3,1,1]	-77.027293
CH_3NC	[6,1]	[6,2]	-132.137112
CH_3OH	[4,0]	[8,4]	-115.305028
CH_3CN	[6,1]	[6,2]	-132.178361
HCN	[2,0,0,0]	[5,0,2,2]	-93.102072
CH_2O	[2,0,0,0]	[5,0,2,3]	-114.112345
CO	[2,0,0,0]	[6,0,2,2]	-113.018224
CO_2	[2,0,0,0,0,1,0,0]	[3,0,1,1,0,3,2,2]	-188.003281
NH_3	[2,0]	[4,2]	-56.319839
N_2O	[4,0,0,0]	[6,0,4,4]	-184.141570
H_2O	[1,0,0,0]	[4,0,1,3]	-76.197095
HF	[1,0,0,0]	[4,0,2,2]	-100.281376
F_2	[1,0,0,0,0,1,0,0]	[2,0,2,2,0,2,2,2]	-199.120916

II. CORE-IONIZATION ENERGIES AND SPECTROSCOPIC FACTORS OF OZONE

Core: $[4,0,0,2]$; Active: $[3,1,2,3]$

TABLE S2. Oxygen K-edge core ionization energies (ω, eV) and spectroscopic factors (S) of ozone calculated using different EOM-DSRG methods. O_C and O_T stand for the central and terminal oxygen atoms, respectively.

Ionization	EOM-DSRG-PT2		EOM-DSRG-PT3		EOM-LDSRG(2)	
	ω	S	ω	S	ω	S
O_T $(1a_1^{-1})$	545.49	1.476	544.64	1.431	544.62	1.435
O_T $(1b_2^{-1})$	545.49	1.476	544.64	1.431	544.62	1.435
O_C $(2a_1^{-1})$	549.17	1.488	548.78	1.473	548.63	1.472
